# Cholesterol 25-hydroxylase suppresses avian reovirus replication by its enzymatic product 25-hydroxycholesterol

**DOI:** 10.3389/fmicb.2023.1178005

**Published:** 2023-06-29

**Authors:** Yuyang Wang, Wei Zuo, Yangyang Zhang, Zongyi Bo, Chengcheng Zhang, Xiaorong Zhang, Yantao Wu

**Affiliations:** ^1^Jiangsu Co-Innovation Center for Prevention and Control of Animal Infectious Disease and Zoonoses, College of Veterinary Medicine, Yangzhou University, Yangzhou, China; ^2^Testing Center, Yangzhou University, Yangzhou, China; ^3^Joint International Research Laboratory of Agriculture and Agri-Product Safety, The Ministry of Education of China, Yangzhou University, Yangzhou, China

**Keywords:** avian reovirus, cholesterol 25-hydroxylase, 25-hydroxycholesterol, virus replication, interferon stimulated genes (ISG)

## Abstract

Avian reovirus (ARV) causing viral arthritis/tenosynovitis and viral enteritis in domestic fowl has significantly threatened on the poultry industry worldwide. ARV is a non-enveloped fusogenic virus that belongs to the Reoviridae family. Previous research revealed that cellular cholesterol in lipid rafts is essential for ARV replication. It has been reported that cholesterol 25-hydroxylase (CH25H) and its product 25-hydroxycholesterol (25HC) have antiviral activities against enveloped viruses. However, few studies characterized the association of non-enveloped viruses with CH25H and the role of CH25H in the regulation of ARV replication. In this study, the expression of chicken CH25H (chCH25H) was found to be upregulated in ARV-infected cells at the early stage of infection. The results of overexpression and knockdown assays revealed that chCH25H has a significant antiviral effect against ARV infection. Furthermore, a 25HC treatment significantly inhibited ARV replication in a dose-dependent manner at both the entry and post-entry stages, and a chCH25H mutant lacking hydroxylase activity failed to inhibit ARV infection. These results indicate that CH25H, depending on its enzyme activity, exerts the antiviral effect against ARV via the synthesis of 25HC. In addition, we revealed that 25HC produced by CH25H inhibits viral entry by delaying the kinetics of ARV uncoating, and CH25H blocks cell–cell membrane fusion induced by the p10 protein of ARV. Altogether, our findings showed that CH25H, as a natural host restriction factor, possessed antiviral activity against ARV targeting viral entry and syncytium formation, through an enzyme activity-dependent way. This study may provide new insights into the development of broad-spectrum antiviral therapies.

## 1. Introduction

Avian reovirus (ARV) belongs to the Orthoreovirus genus of the Reoviridae family (Mertens, 2004). Avian reovirions are non-enveloped icosahedral particles with a segmented double-stranded RNA (dsRNA) genome. The genome encodes 12 viral proteins including eight structural (*λ*A, *λ*B, *λ*C, *μ*A, *μ*B, *σ*A, *σ*B, and *σ*C) and four nonstructural proteins (*μ*NS, *σ*NS, p10, and p17) (Benavente and Martinez-Costas, 2007). ARV has been implicated in several severe diseases and conditions that cause significant economic losses in the poultry industry, including viral arthritis/tenosynovitis, chronic respiratory disease, runting-stunting syndrome, and immunosuppression (Jones and Kibenge, 1984; Jones, 2000; Neelima et al., 2003).

Understanding the host-virus response is critical for preventing viral infections. Interferon-stimulated genes (ISGs) are the effectors that are induced during interferon (IFN) responses and play critical roles in innate immune defense against viral infections (Sen and Sarkar, 2007; Poynter and DeWitte-Orr, 2016; Raniga and Liang, 2018). Cholesterol 25-hydroxylase (CH25H) is an interferon-induced enzyme that belongs to the ISGs. It can catalyze the oxidation of cholesterol to 25-hydrocholesterol (25HC), a minor side-chain oxysterol (Park and Scott, 2010; Liu et al., 2013). The chCH25H gene, located on chicken chromosome 6, is conserved across different species and is stimulated by interferons. This gene is responsible for encoding an enzyme that is comprised of 274 amino acids, sharing homology with the human CH25H protein (Lund et al., 1998; Holmes et al., 2011). While information concerning CH25H in chickens is limited, there is an abundance of research available on other species. Overall, the primary function of CH25H is to catalyze the production of 25HC from cholesterol by using O_2_ as an additional substrate and NADPH as a co-factor, in order to reduce cholesterol accumulation (Liu et al., 2013; Zang et al., 2020; Zhao et al., 2020). It is a multi-pass transmembrane endoplasmic reticulum (ER)-associated enzyme with the N-terminus located outside the ER (Lund et al., 1998; Cyster et al., 2014). The CH25H contains three clusters of histidine residues (histidine box), and the third histidine box (residues 238–244) has been reported to be essential for catalytic activity (Holmes et al., 2011; Chen et al., 2014). It has been reported that CH25H can be rapidly induced in a variety of mammalian tissues including liver, heart, muscle, brain, kidney, and lung. Tissues with resident macrophage populations show the highest levels of CH25H induction (Bauman et al., 2009; Park and Scott, 2010).

Recently, CH25H and its product 25HC have been reported to exert multifaceted functions to regulate cholesterol homeostasis, antivirus process, inflammatory response, and cell survival (Cao et al., 2020; Zhao et al., 2020). Many studies have shown that CH25H and 25HC exhibit broad antiviral activities against a variety of enveloped viruses, such as the human immunodeficiency viruses (HIV), hepatitis C virus (HCV), severe acute respiratory syndrome coronavirus-2 (SARS-CoV-2), Zika virus (ZIKV), vesicular stomatitis virus (VSV), murine cytomegalovirus (MCMV), Lassa fever virus (LASV), Ebola virus, and influenza virus (Liu et al., 2013; Lembo et al., 2016; Shrivastava-Ranjan et al., 2016; Zhao et al., 2020; Davidson and Rotondo, 2021). Moreover, CH25H and 25HC exert antiviral effector function via multiple mechanisms (Zhao et al., 2020). For enveloped viruses, the mechanism of this effect primarily involves altering cholesterol homeostasis and membrane physiology to impede virus-cell membrane fusion, viral morphogenesis, and egress (such as HIV, HCV, SARS-CoV-2) (Liu et al., 2013; Kusuma et al., 2015; Zang et al., 2020; Wang S. et al., 2020), disturbing post-translational modification of proteins (such as SARS-CoV-2, LASV) (Shrivastava-Ranjan et al., 2016; Mao et al., 2022) and regulating of inflammation and immunity during virus infection (such as ZIKV, influenza virus) (Bauman et al., 2009; Gold et al., 2014). Besides enzyme the activity-dependent antiviral mechanism, some antiviral functions of CH25H may not involve 25HC production (Dong et al., 2018; Ke et al., 2019; Lv et al., 2019). For instance, Dong et al. found that CH25H inhibited PRRSV infection not only by producing 25HC to inhibit viral penetration, but also by degrading nonstructural protein 1 alpha (nsp1α) of PRRSV through the ubiquitin-proteasome pathway (Dong et al., 2018).

Contrary to the well-studied enveloped viruses, there is far fewer data on the antiviral effect of CH25H against non-enveloped viruses, which lack outer lipid membranes. Initially, it was thought that CH25H-induced 25HC would be ineffective against non-enveloped viruses because the non-enveloped virus can directly enter the cell without the fusion process. Take the case of adenovirus, a non-enveloped virus, was demonstrated not to be affected by 25HC (Civra et al., 2014). However, recent research has shown that 25HC has potent antiviral activity against some non-enveloped viruses, including poliovirus, human rhinovirus (HRhV), human papillomavirus type 16 (HPV-16), human rotavirus (HRoV), and mammalian orthoreovirus (MRV). For these non-enveloped viruses, 25HC exerts antiviral activity by targeting members of oxysterol binding protein (OSBP) (e.g., Poliovirus, HRhV), targeting essential intracellular events of virus replication cycle (e.g., HPV-16), and sequestering viral particles into late endosomes (e.g., HRoV) (Civra et al., 2014, 2018; Roulin et al., 2014; Lembo et al., 2016; Doms et al., 2018). Nonetheless, the functions of CH25H on non-enveloped viruses are only now being explored, and the underlying mechanisms are still elusive. A more recent study suggested that 25HC production by CH25H probably reduces the efficiency of cellular entry of MRV virions (Doms et al., 2018). Unlike MRV, ARV is a member of the group of fusogenic reoviruses, which are the only non-enveloped viruses known to cause cell–cell fusion (Shmulevitz and Duncan, 2000). Additionally, our previous study showed that cellular cholesterol in lipid rafts plays a critical role in ARV replication at both the entry stage and the post-entry stages (Wang Y. et al., 2020). The unusual fusogenic ability of ARV and the importance of cholesterol homeostasis in ARV infection deserve attention. Thus, the functions and underlying mechanisms of CH25H on ARV remain to be further characterized.

In this study, we identified that ARV infection triggered the induction of the interferon-stimulated protein CH25H in the antiviral innate immunity, and the CH25H possessed antiviral activity against ARV by its enzymatic product 25HC through an enzyme activity-dependent way. Moreover, we provided evidence that CH25H and 25HC inhibit ARV infection by delaying the kinetics of ARV uncoating and blocking ARV-induced membrane fusion. These findings suggest that 25HC has the potential as a natural antiviral agent to combat ARV, and may help to develop novel antiviral strategies.

## 2. Materials and methods

### 2.1. Cells and viruses

DF-1 and HD11 cells were maintained in Dulbecco’s modified Eagle’s medium (DMEM, Gibco, Grand Island, United States) supplemented with 10% fetal bovine serum (FBS) (Gibco) at 37°C in a humidified incubator with 5% CO_2_. The ARV strain GX/2010/1 (GenBank accession no. KJ476699) was propagated and used as previously described (Duan et al., 2015; Wang Y. et al., 2020).

### 2.2. Reagents and antibodies

25-Hydroxycholesterol (25HC) was purchased from MedChemExpress (Monmouth Junction, United States). Polyinosinic–polycytidylic acid [poly (I:C)] was purchased from Sigma-Aldrich (St. Louis, United States) Wright-Giemsa Stain Kit was purchased from Baso Biotechnology (Zhuhai, China). Rabbit monoclonal antibodies against HA-Tag and Myc-Tag were purchased from Cell Signaling Technology (Danvers, United States). Mouse polyclonal antibodies against *σ*B and *μ*NS of ARV were prepared in our laboratory. Mouse monoclonal antibodies against GAPDH and *β*-actin were prepared from Proteintech (Rosemont, United States). Horseradish peroxidase (HRP)-conjugated goat anti-mouse IgG/goat anti-rabbit IgG were purchased from Thermo Fisher Scientific (Waltham, United States). radioimmunoprecipitation assay (RIPA) buffer, phenylmethylsulfonyl fluoride (PMSF), and SDS-PAGE loading buffer were purchased from Beyotime Biotechnology (Shanghai, China). The viability of DF-1 cells after 25HC treatment was determined using the Cell Counting Kit 8 (APExBIO, Houston, United States).

### 2.3. Plasmids

The chicken CH25H gene (GenBank accession NM_001277354.1) was synthesized by GenScript (Nanjing) Co., Ltd. (Nanjing, China) and cloned into the pcDNA-3.1(+) vector (Invitrogen) to generate Myc-tagged expression plasmid pcDNA3.1-CH25H-Myc. An expression vector containing genes encoding Myc-tagged chCH25H mutant (H242Q and H243Q) that lacks hydroxylase activity, named as pcDNA3.1-CH25H-M-Myc, was constructed by site-directed mutagenesis according to the manufacturer’s instructions (TransGen Biotech, Shanghai, China). The primers used for the construction of plasmids expressing full-length wild-type and mutant CH25H are listed in Table 1. All constructed plasmids were verified by sequencing. Plus, the pcDNA3.1-p10-HA plasmids were prepared in our laboratory.

**Table 1:** The primer sequences for construction of the expression vector containing genes encoding Myc-tagged chCH25H mutant (H242Q and H243Q) by site-directed mutagenesis.

Primer name	Primer sequence (5′-3′)
chCH25H-M-F	TGGTATGGAGGAGCACCGCAACAAGATCTCCATCATCTG
chCH25H-M-R	TTGTTGCGGTGCTCCTCCATACCAACCGAAAGGCACAAG

### 2.4. CH25H expression and virus challenge

DF-1 cells were transfected with respective constructs (pcDNA3.1-CH25H-Myc or pcDNA3.1) at about 70% confluence, using TransIntro EL Transfection Reagent (TransGen Biotech, Beijing, China), as indicated by the manufacturer. After 24 h post-transfection, the cells were infected with ARV at an MOI of 1. The ARV replication was analyzed by qRT-PCR and Western blotting at 24 h postinfection (hpi). In addition, the overexpression of CH25H mutant and virus challenge were carried out as described above.

### 2.5. 25HC treatment and ARV infection assay

To investigate whether 25HC restricts ARV infection at the viral entry stage, DF-1 cells were mock-treated or pre-treated with 25HC (2.5, 5 or 10 μM) at 37°C for 12 h, followed by infecting cells with 1 MOI ARV at 37°C for 1 h (Doms et al., 2018). The inoculum was removed, and the bound but unpenetrated virions were inactivated by citrate buffer as previously described (Wang Y. et al., 2022). The ARV replication levels in cells were evaluated using qRT-PCR and Western blotting at 24 hpi.

To determine whether 25HC affects ARV replication at the viral post-entry stage, DF-1 cells were infected with 1 MOI ARV at 37°C for 1 h, followed by treated with 25HC (2.5, 5, or 10 μM) or mock-treated at 37°C for 24 h. Then, the cells were harvested and the ARV replication levels were measured using qRT-PCR and Western blotting.

### 2.6. Small interfering RNA (siRNA) assay

Three pairs of siRNAs specifically targeting chCH25H and a non-specific control siRNA (negative control, NC) were designed and synthesized by GenePharma (Shanghai, China). The DF-1 cells growing in 6-well plates were transfected with siRNAs or NC using TransIntro EL Transfection Reagent (TransGen Biotech), following the manufacturer’s instructions. At 36 h post-transfection, the knockdown efficiency of CH25H was detected by qRT-PCR. After CH25H was reduced, the cells were infected with ARV at an MOI of 1, and ARV replication was evaluated by qRT-PCR, Western blotting, and virus titration at 24 hpi. The primer sequences used for interference experiments were listed in Table 2.

**Table 2:** The primer sequences for siRNA assay.

Primer name	Primer sequence (5′-3′)
chCH25H-siRNA-NC-F	UUCUCCGAACGUGUCACGUTT
chCH25H- siRNA-NC-R	ACGUGACACGUUCGGAGAATT
chCH25H-siRNA1-F	GCUAUCCAACUCUUGGAAUTT
chCH25H-siRNA1-R	AUUCCAAGAGUUGGAUAGCTT
chCH25H-siRNA2-F	GCUCUUACUACACAGUAUUTT
chCH25H-siRNA2-R	AAUACUGUGUAGUAAGAGCTT
chCH25H-siRNA3-F	GCAUCAUGAUCUCCAUCAUTT
chCH25H-siRNA3-R	AUGAUGGAGAUCAUGAUGCTT

### 2.7. Reverse transcription and real-time PCR

Cells were lysed by the TRIzol reagent RNA kit (CWbio, Beijing, China), and total RNA was extracted and then reverse transcribed into cDNA using M-MLV reverse transcriptase (TransGen Biotech, Beijing, China), according to the manufacturer’s instructions. Quantitative real-time PCR (qPCR) experiments were performed in triplicate using PowerUp™ SYBR™ Green Master Mix (Thermo Fisher Scientific) on an Applied Biosystems 7,500 Fast Real-Time PCR System as described previously (Wang Y. et al., 2022). The GAPDH/*β*-actin was used as the endogenous control, and relative gene expression analysis was performed using the 2^*−*∆∆CT^ method. All primers used for qPCR are listed in Table 3.

**Table 3:** The primer sequences for qRT-PCR.

Primer name	Primer sequence (5′-3′)
*σ*C-F	CGTATCATTCACCCGCGATT
*σ*C-R	TGTTCGCTGTACCATCACCT
*μ*NS-F	CGTGTGGAAGCGTTAAACCA
*μ*NS-R	TCATCACGCTCGTTCAGGTA
CH25H-F	AATCCAGCCGCAGAGCTATC
CH25H-R	CAGCTCTGGAGCTAYCACCG
IFNA-F	ATGCCACCTTCTCTCACGAC
IFNA-R	AGGCGCTGTAATCGTTGTCT
IFNB-F	ACCAGGATGCCAACTTCT
IFNB-R	TCACTGGGTGTTGAGACG
*β*-actin-F	ATTGTCCACCGCAAATGCTTC
*β*-actin-R	AAATAAAGCCATGCCAATCTCGTC
GAPDH-F	GCACTGTCAAGGCTGAGAACG
GAPDH-R	GATGATAACACGCTTAGCACCAC

### 2.8. Enzyme-linked immunosorbent assay (ELISA)

The levels of chicken IFNA, chicken IFNB, and 25HC in cell cultures were measured by ELISA following the manufacturer’s instructions. The ELISA kits for chicken IFNA, IFNB, and 25HC were obtained from Jiangsu Meimian Industrial Co. Ltd. (Yancheng, China).

### 2.9. Virus titration

The ARV-infected cells in the relevant experiments were harvested by freeze-thawing and quantified by virus titration. In the relevant experiments, the progeny virus titer of ARV in the cell lysates was determined by calculating the 50% tissue culture infective dose (TCID_50_) of CEF cells, using the Reed-Muench method.

### 2.10. SDS-PAGE and Western blotting analyses

SDS-PAGE and Western blotting analyses were carried out as described elsewhere (Wang Y. et al., 2020). The cells for protein detection were extracted in RIPA lysis buffer supplemented with 1 mM PMSF. Proteins were resolved in 10% Bis-Tris gels and transferred to PVDF membranes (Pall Corporation, New York, United States). The membranes were blocked and followed by incubation with the respective primary antibodies and appropriate HRP-conjugated secondary antibodies. The protein bands were detected with developed using an enhanced chemiluminescence (ECL) detection system (Tanon Imager, Shanghai, China).

### 2.11. Measurement of the kinetics of ARV outer capsid proteolysis

The cells grown in 6-well plates were mock-treated, or pre-treated with 5 μM 25HC for 12 h at 37°C, and chilled at 4°C for 1 h. Then, the cells were adsorbed with 100 MOI ARV at 4°C for 1 h. After the unbound virus was removed by washing with PBS, the cells were further incubated with prewarmed DMEM supplemented with 2% FBS at 37°C for 0, 60, 90, 120, or 150 min. The cells were scraped and lysed, and the proteolysis of ARV outer capsid protein was detected by the polyclonal antibodies against *σ*B using Western blotting. ARV-cell binding assays were carried out as described previously (Wang Y. et al., 2020).

### 2.12. Cell-to-cell fusion assay

Cell-to-cell fusion assay based on the expression of p10 was performed *in vitro* without ARV infection. DF-1 cells grown in 24-well culture plates were co-transfected with the plasmids pcDNA3.1-p10-HA and pcDNA3.1-CH25H-Myc/pcDNA3.1-CH25H-M-Myc/pcDNA3.1 empty vector, respectively. At 48 h post-transfection, the cells were fixed and stained with Wright-Giemsa. The stained cells were observed using an inverted microscope (Olympus, Tokyo, Japan), and the syncytium formation was determined by the average number of syncytia from five random fields using a × 10 objective.

### 2.13. Statistical analysis

All statistical tests were performed using GraphPad Prism 8.0 (GraphPad Software, San Diego, United States). The data are expressed as the mean ± standard deviation. Statistical comparisons of two independent groups were performed using Student’s *t-*test, and multiple comparisons were made using one-way ANOVA (*, *p* < 0.05; **, *p* < 0.01).

## 3. Results

### 3.1. ARV infection induces endogenous CH25H expression

**Figure 1:**
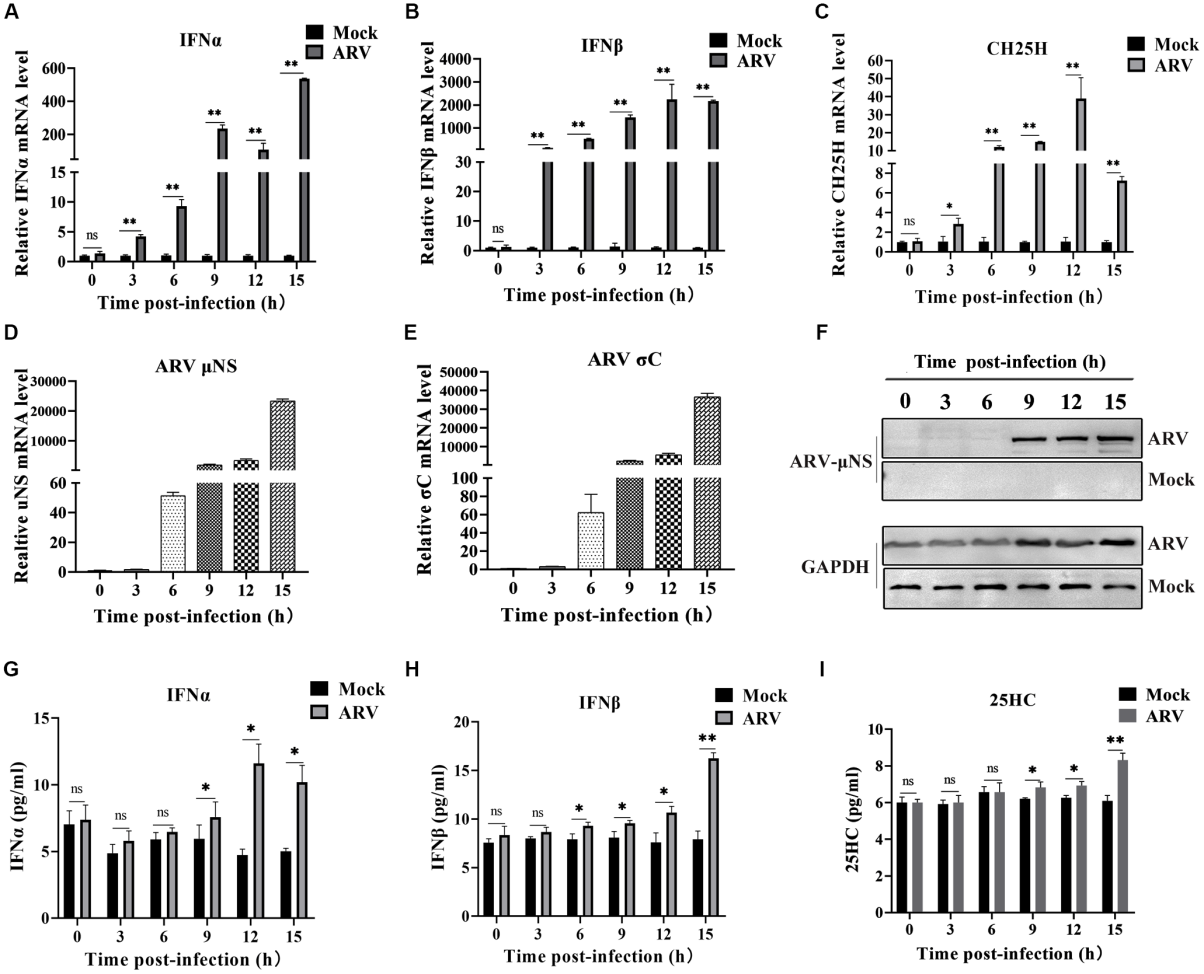
ARV infection induces endogenous CH25H expression. HD11 cells were mock infected or infected with ARV strain GX/2010/1 at an MOI of 1. The mRNA expression levels of IFNA **(A)**, IFNB **(B)**, CH25H **(C)**, ARV *μ*NS **(D)**, and *σ*C **(E)** genes were determined by qRT-PCR at the indicated time points. GAPDH was used as the endogenous control, and relative gene expression analysis was performed using the 2^*−*∆∆CT^ method. **(F)** The protein levels of ARV μNS was analyzed by Western blotting. **(G)** IFNA protein levels were measured by ELISA. **(H)** IFNB protein levels were measured by ELISA. **(I)** The levels of 25HC were analyzed by ELISA. Results are presented as means ± SD from three independent experiments. Significance was determined by Student’s *t-*test (ns, not significant; *, *p* < 0.05; **, *p* < 0.01).

We investigated the expression dynamics of endogenous CH25H after ARV infection in HD11 cells, which is a chicken macrophage cell line, considering that CH25H is an interferon-induced enzyme that belongs to the ISGs. HD11 cells were infected with ARV at an MOI of 1. The mRNA levels of IFNA, IFNB, CH25H, ARV *σ*C and *μ*NS genes were quantified with qRT-PCR at the indicated time points (0, 3, 6, 9, 12, and 15 hpi). As presented in Figures 1A,B, the expression of IFNA and IFNB were significantly upregulated starting at 3 hpi. The expression of CH25H mRNA was significantly upregulated starting at 3 hpi, increased by 10-fold at 6 hpi, peaked by about 35-fold at 12 hpi, and decreased at 15 hpi (Figure 1C). The overall trend of the expression level of CH25H was consistent with the expression levels of IFNA and IFNB after ARV infection. The kinetics of ARV replication in HD11 cells after infection are shown in Figures 1D,E. The expression levels of the ARV *σ*C and *μ*NS genes were upregulated at 3 hpi, surged starting at 6 hpi, and dramatically increased with the progress of virus infection (Figures 1D–F). Changes in the protein levels of IFNA, IFNB, and the change of the production of 25HC during ARV infection were detected by ELISA. The trends were consistent with the transcription levels of the corresponding genes (Figures 1G–I). Furthermore, the HD11 cells and DF-1 cells were transfected with poly (I:C) (2 μg/mL), a type I IFN inducer. The results show that chCH25H is an ISG effector protein in host cells, the mRNA levels of chCH25H, IFNA and IFNB were significantly upregulated after treatment with poly (I:C) in HD11 cells and DF-1 cells (Supplementary Figure S1). Taken together, these results indicated that ARV infection triggered the induction of CH25H at the early stage of infection.

### 3.2. Overexpression of chCH25H dramatically decreases ARV replication and knockdown of chCH25H by siRNA promotes ARV infection

**Figure 2:**
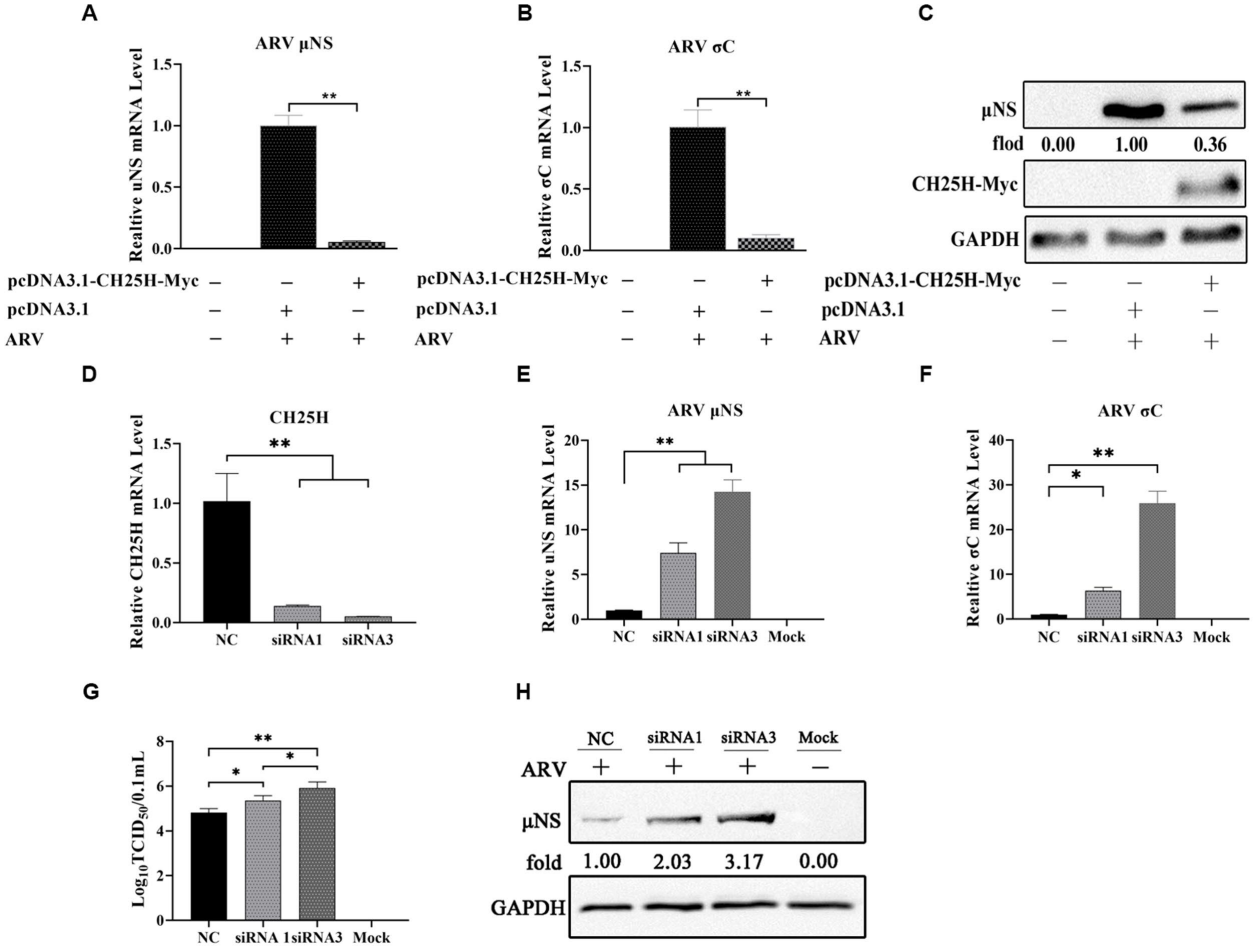
The overexpression of chCH25H dramatically decreases ARV replication and the knockdown of chCH25H by siRNA promotes ARV infection. **(A–C)** DF-1 cells were mock-transfected or transfected with a construct expressing Myc-tagged chCH25H. At 24 h post-transfection, the cells were then infected with ARV at an MOI of 1 for 24 h. **(A)** The mRNA expression levels of the ARV-*μ*NS gene were determined by qRT-PCR. **(B)** The mRNA expression levels of ARV-*σ*C gene were determined by qRT-PCR. **(C)** The protein levels of ARV *μ*NS were analyzed by Western blotting. **(D–H)** The DF-1 cells were transfected with siRNAs or negative control. At 24 h post-transfection, the cells were then infected with ARV at an MOI of 1 for 24 h. **(D)** The knockdown efficiency of CH25H was detected by qRT-PCR at 36 h post-transfection. **(E)** The mRNA expression levels of the ARV-*μ*NS gene were determined by qRT-PCR. **(F)** The mRNA expression levels of the ARV-*σ*C gene were determined by qRT-PCR. **(G)** The number of infectious virus particles was quantified by TCID_50_ analysis. **(H)** The protein levels of ARV μNS were analyzed by Western blotting. Results are presented as means ± SD from three independent experiments. Significance was determined by Student’s *t-*test (*, *p* < 0.05; **, *p* < 0.01).

To investigate whether CH25H could inhibit ARV replication, we exogenously expressed myc-tagged chCH25H in DF-1 cells prior to ARV infection. The vectors pcDNA3.1-CH25H-Myc and pcDNA3.1 were transiently transfected into DF-1 cells, respectively. After 24 h post-transfection, the cells were infected with an equal amount of ARV at an MOI of 1. The cell lysates were collected at 24 hpi and the ARV replication was evaluated by qRT-PCR and Western blotting. Compared with empty vector controls, the mRNA expression levels of the *μ*NS and *σ*C genes in overexpressed chCH25H cells were substantially reduced to 5 and 10%, respectively (Figures 2A,B). Moreover, the level of ARV *μ*NS protein in the cells that overexpressing chCH25H was also dropped to 36% compared to controls (Figure 2C). These data indicated that overexpression of chCH25H significantly suppressed ARV infection.

**Figure 3:**
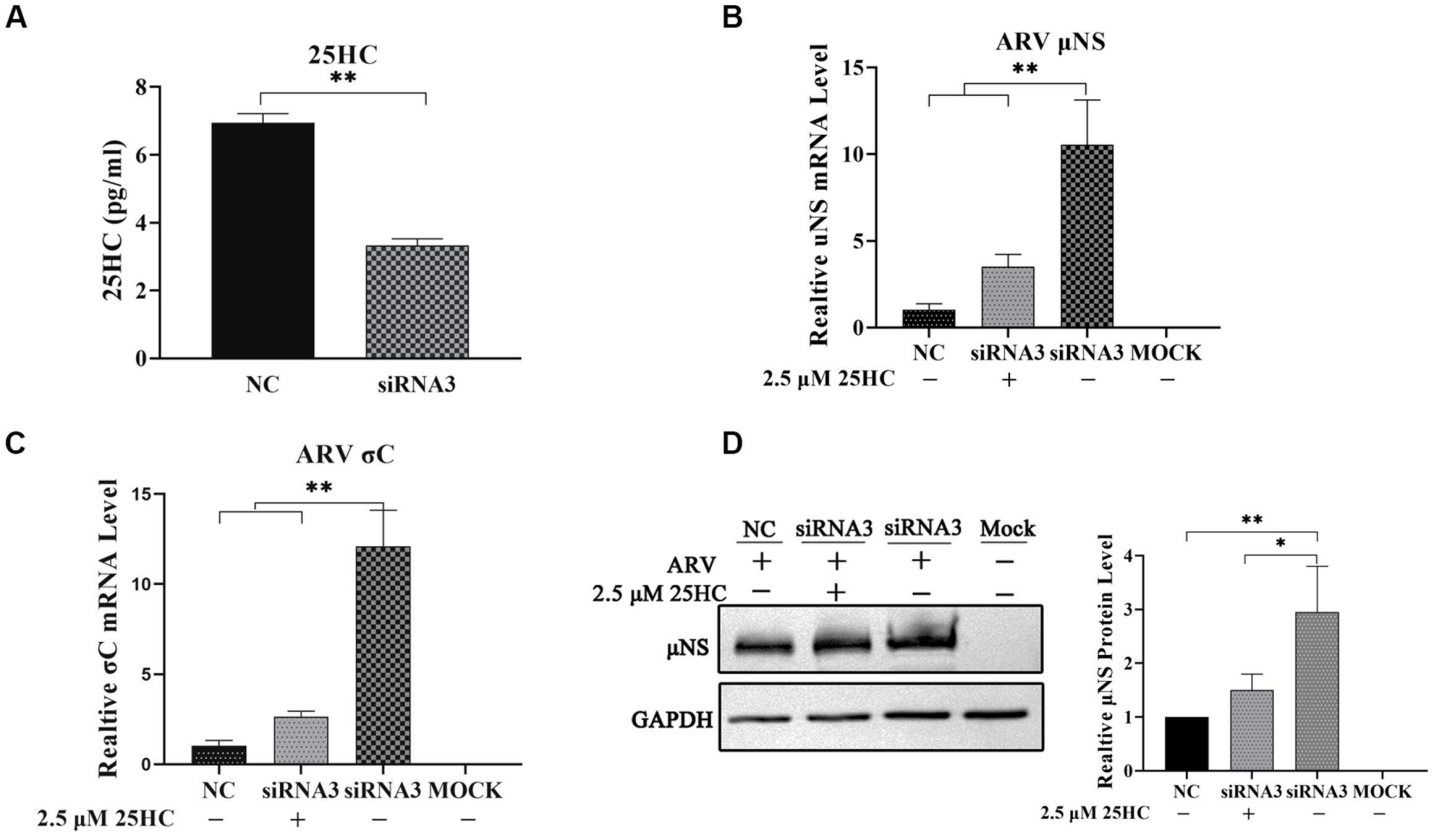
The knockdown of chCH25H by siRNA decreases production of 25HC and the extra addition of 25HC rescued the antiviral effect of CH25H in the cells transfected with siRNA. **(A)** The levels of 25HC were analyzed by ELISA following siRNA treatment. **(B)** The mRNA expression levels of the ARV-*μ*NS gene were determined by qRT-PCR. **(C)** The mRNA expression levels of ARV-*σ*C gene were determined by qRT-PCR. **(D)** The protein levels of ARV *μ*NS were analyzed by Western blotting. Results are presented as means ± SD from three independent experiments. Significance was determined by Student’s *t-*test (*, *p* < 0.05; **, *p* < 0.01).

To further define the antiviral ability of CH25H against ARV, we knocked down the levels of the chCH25H in DF-1 cells using siRNA. The knockdown efficiency of siRNA1, siRNA2, and siRNA3 targeting chCH25H was determined by qRT-PCR at 48 h post-transfection. SiRNA1 and siRNA3 presented the most significant down-regulation of chCH25H (Supplementary Figure S2) and were subsequently chosen for interference experiments. DF-1 cells were transfected with siRNA1, siRNA3, or NC siRNA, and infected with ARV at an MOI of 1 at 24 h post-transfection. ARV replication was evaluated by qRT-PCR, Western blotting, and TCID_50_ assay at 24 hpi. As expected, the siRNAs dramatically reduced chCH25H levels in DF-1 cells, and chCH25H RNA levels decreased by more than 85%, relative to that in cells transfected with NC siRNA (Figure 2D). The expression levels of *σ*C mRNA and *μ*NS mRNA from the siRNA1 group was both significantly upregulated by >5-fold compared to NC (Figure 2E). In the siRNA 3 group, the level of *σ*C mRNA was significantly upregulated by approximately 25-fold, and the level of *μ*NS mRNA was significantly upregulated by approximately 14-fold (Figure 2F). The results of Western blotting analysis and TCID_50_ analysis were consistent with those of qRT-PCR (Figures 2G,H). Furthermore, the production of 25HC was quantified following siRNA treatment, and a rescue experiment of the RNAi effect by the addition of 25HC was also performed to validate our siRNA data. In briefly, the knockdown of chCH25H by siRNA decreases production of 25HC and the extra addition of 25HC rescued the antiviral effect of CH25H in the cells transfected with siRNA (Figure 3). Thus, these data suggested that the knockdown of chCH25H significantly promoted ARV infection in DF-1 cells.

### 3.3. 25HC inhibits ARV replication in a dose-dependent manner beyond the viral initial entry

**Figure 4:**
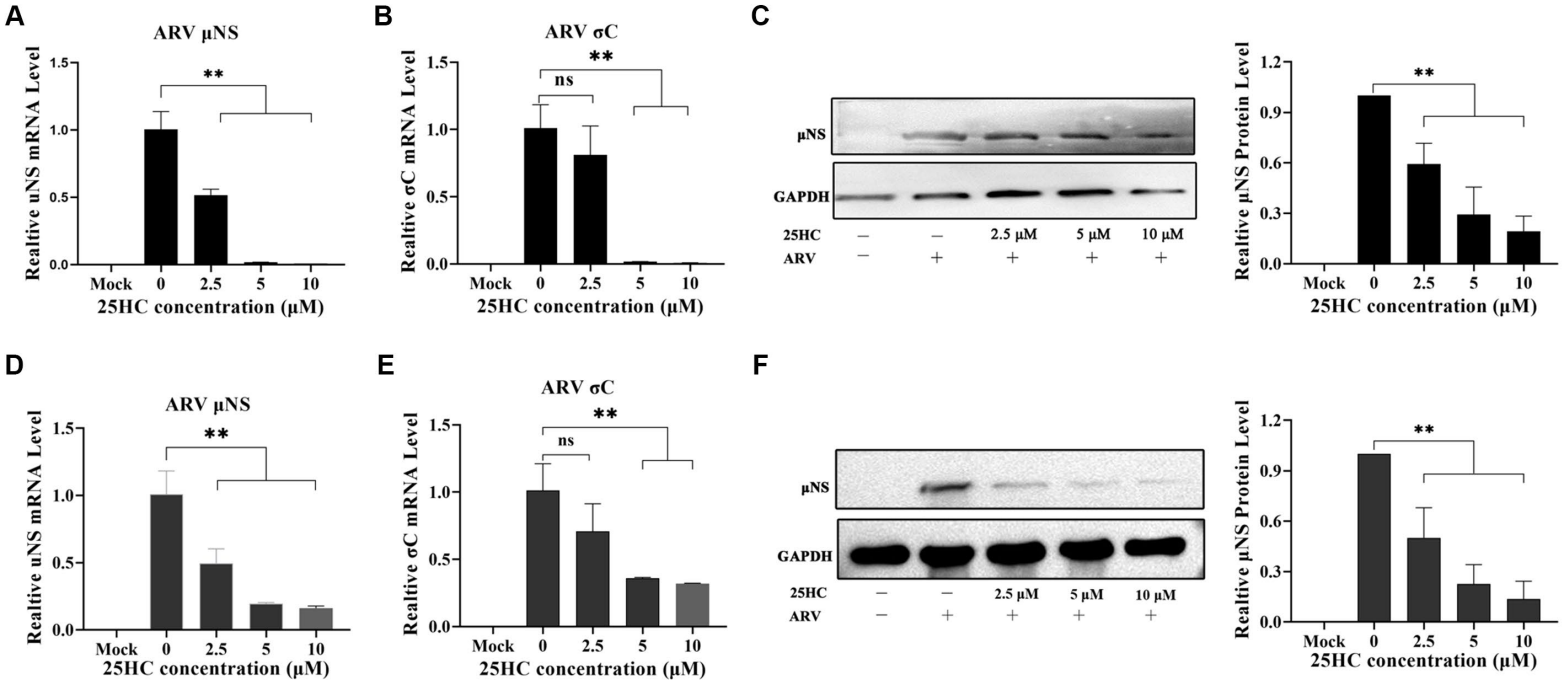
25HC inhibits ARV replication in a dose-dependent manner beyond the viral initial entry. The DF-1 cells were mock-treated or treated with the indicated concentrations (2.5, 5, and 10 mM) of 25HC at the viral entry and post-entry stages. **(A–C)** The effects of 25HC treatment before ARV infection. **(A)** The mRNA expression levels of the ARV-*μ*NS gene were determined by qRT-PCR. **(B)** The mRNA expression levels of ARV-*σ*C gene were determined by qRT-PCR. **(C)** The level of the ARV *μ*NS protein was analyzed by Western blotting. **(D–F)** The effects of 25HC treatment after ARV infection. **(D)** The mRNA expression levels of the ARV-*μ*NS gene were determined by qRT-PCR. **(E)** The mRNA expression levels of the ARV-*σ*C gene were determined by qRT-PCR. **(F)** The level of the ARV *μ*NS protein was analyzed by Western blotting. Results are presented as means ± SD from three independent experiments. Significance was determined by one-way ANOVA (ns, not significant; *, *p* < 0.05; **, *p* < 0.01).

25-HC is one of the oxidation products of cholesterol synthesized by CH25H. Before evaluating the effect of 25HC on ARV infection, the cytotoxicity of 25HC was determined in DF-1 cell lines using CCK-8. After incubation with 25HC at concentrations of less than 10 mM for 24 h, no obvious cytotoxicity was observed in DF-1 cells (data not shown). To investigate the suppressive effect of 25HC against ARV, the DF-1 cells were mock-treated or treated with the indicated concentrations (2.5, 5, and 10 mM) of 25HC at the viral entry and post-entry stages. As presented in Figures 4A–C, pre-treatment of DF-1 cells with increasing concentrations of 25HC prior to ARV infection, reduced *μ*NS and *σ*C expression in a dose-dependent manner. We further tested whether the presence of 25HC restricts ARV replication after viral entry has taken place. The addition of 25HC in ARV-infected cells at 1 h post-entry significantly inhibited ARV replication, also in a dose-dependent manner (Figures 4D–F). Altogether, the results suggested that 25HC restricted ARV infection at both the entry and post-entry stages of the ARV replication cycle.

### 3.4. The chCH25H mutant (H242Q and H243Q) that lacks hydroxylase activity fails to inhibit ARV infection

We tested whether the hydroxylase activity of chCH25H is essential for the efficient suppression of ARV replication. A CH25H catalytic mutant (H242Q and H243Q) was generated to investigate a potential 25HC-independent antiviral function of chCH25H. In addition, 25HC was quantified using an ELISA kit to measure the enzyme activity of chCH25H and chCH25H mutant. The results show that the chCH25H mutant (H242Q and H243Q) indeed loses the ability to produce 25HC (Supplementary Figure S3).

**Figure 5:**
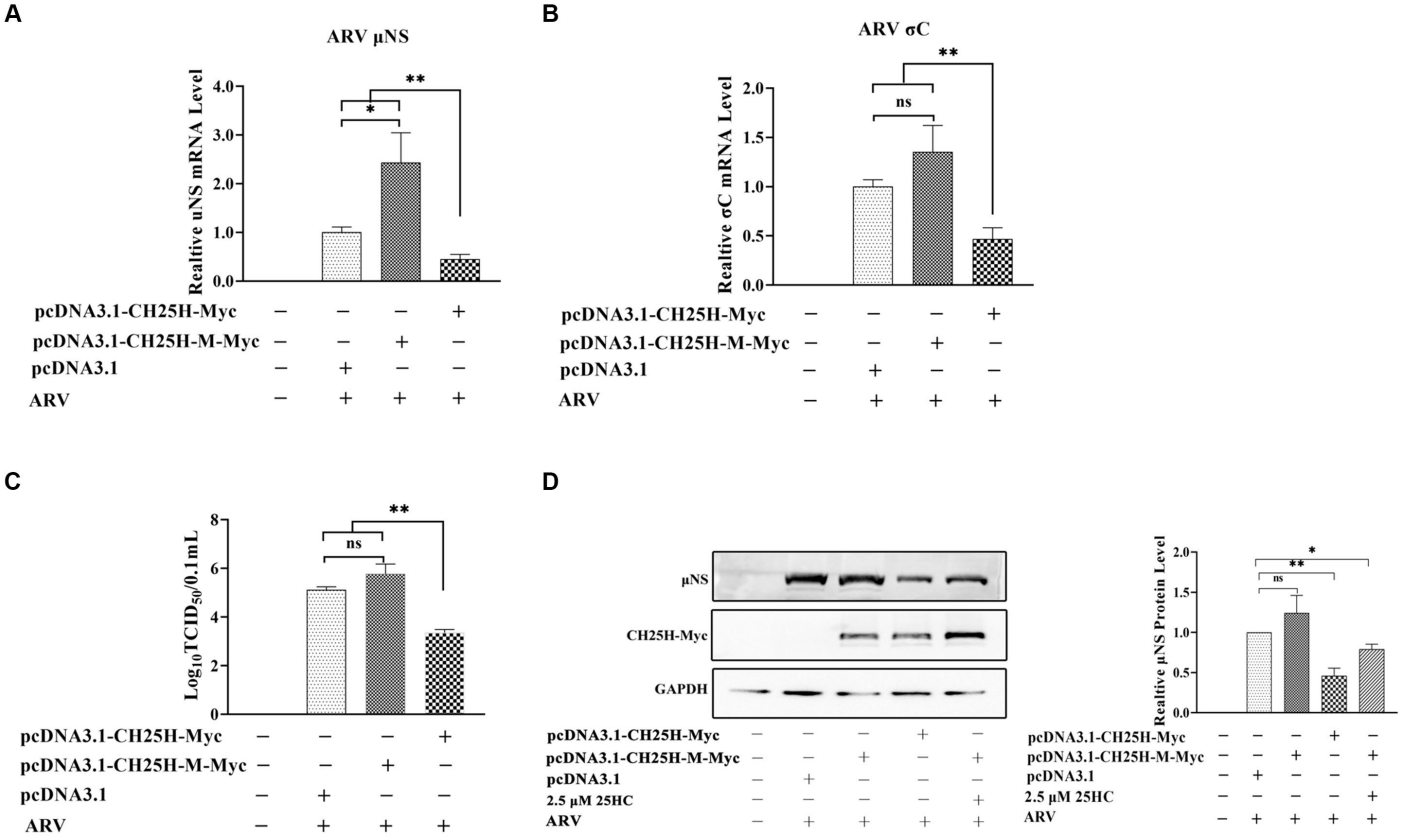
The chCH25H mutant that lacks hydroxylase activity failed to inhibit ARV infection. DF-1 cells were transfected with wild-type chCH25H, enzymatic mutant chCH25H, or an empty vector for 24 h, and infected with ARV at an MOI of 1 for 24 h. **(A)** The mRNA expression levels of the ARV-*μ*NS gene were determined by qRT-PCR. **(B)** The mRNA expression levels of the ARV-*σ*C gene were determined by qRT-PCR. **(C)** The number of infectious virus particles was quantified by TCID_50_ analysis. **(D)** The levels of wild-type chCH25H, enzymatic mutant chCH25H, and the ARV *μ*NS protein were analyzed by Western blotting. Results are presented as means ± SD from three independent experiments. Significance was determined by Student’s *t-*test (ns, not significant; *, *p* < 0.05; **, *p* < 0.01).

DF-1 cells were transfected with the wild-type chCH25H, enzymatic mutant chCH25H, or an empty vector for 24 h, and then infected with ARV at an MOI of 1. The ARV mRNA and protein expression levels, and virus titers were quantified at 24 hpi (Figure 5). The mRNA levels of the *μ*NS and *σ*C genes, *μ*NS protein levels, and ARV titers in the wild-type chCH25H group were significantly decreased, compared to the empty vector control group as expected. However, in the mutant chCH25H group, *μ*NS and *σ*C mRNA levels, and ARV titers were significantly higher than those in the wild-type chCH25H group. The ARV *μ*NS protein levels of the mutant chCH25H were also much higher than those of the wild-type chCH25H group. In addition, when comparing the mutant group with the empty vector group, although the overexpression of mutant chCH25H increased the *μ*NS transcription and protein expression, the overexpression of mutant chCH25H had no significant effect on ARV *σ*C transcription and progeny virus production (Figure 5). Furthermore, the extra addition of 25HC sufficiently complemented the lost activity of the chCH25H mutant (Figure 5D). These results revealed that the chCH25H mutant lost the ability to inhibit ARV replication.

### 3.5. 25HC delays proteolysis of ARV outer-capsid protein *σ*B

**Figure 6:**
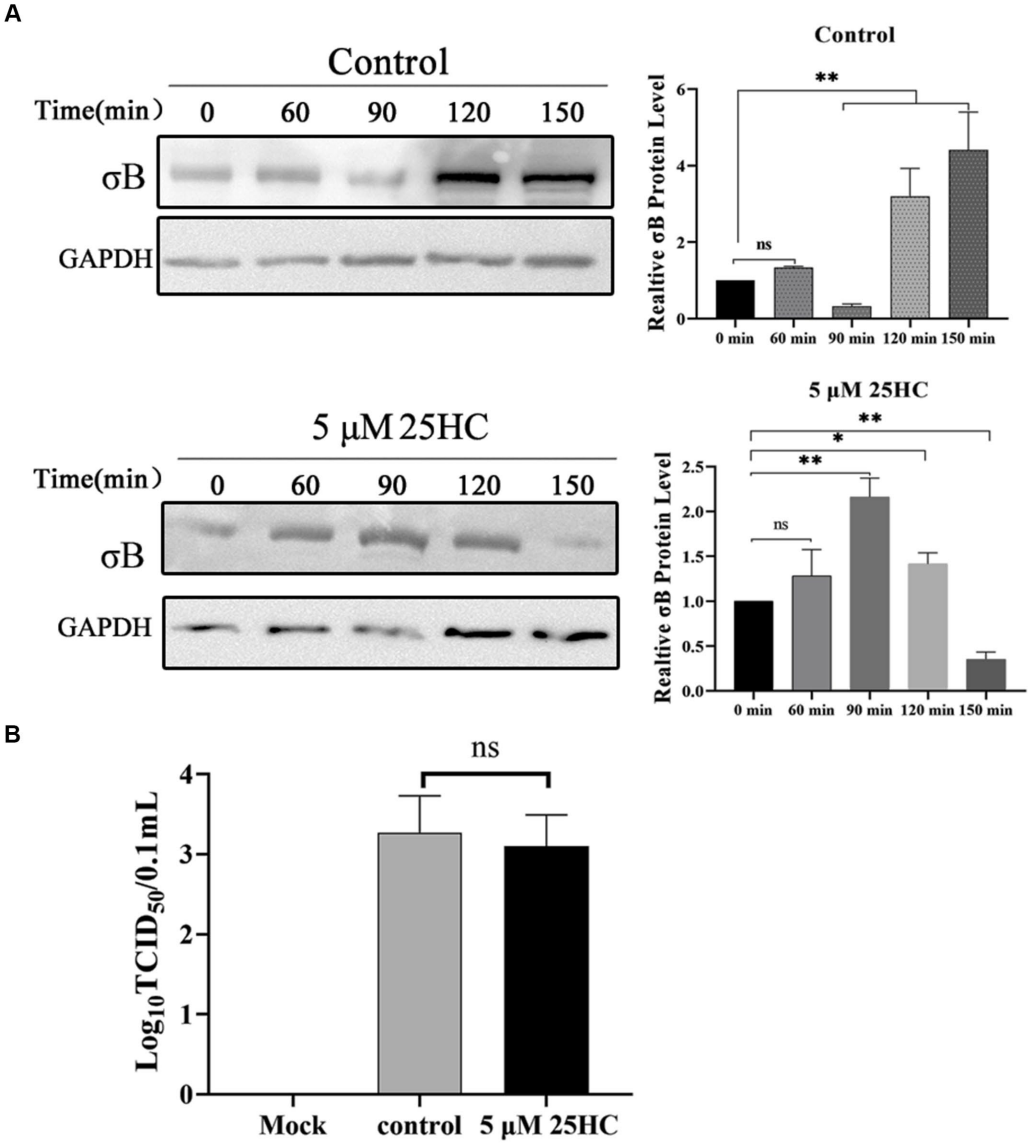
25HC delays outer-capsid protein *σ*B proteolysis of ARV. **(A)** DF-1 cells were pretreated with 5 μM 25HC or mock treated for 12 h, followed by incubation with 100 MOI ARV at 4°C for 1 h. After the unbound virus was removed, the cells were further incubated at 37°C for 0, 60, 90, 120, or 150 min. The *σ*B protein levels in cell lysates were tested by Western blotting at the indicated time points. **(B)** DF-1 cells were pretreated with 5 μM 25HC or mock treated for 12 h, followed by incubation with 100 MOI ARV at 4°C for 1 h. Virions that were attached to cells were quantified by TCID_50_ analysis. Results are presented as means ± SD from three independent experiments. Significance was determined by Student’s *t-*test (ns, not significant; *, *p* < 0.05; **, *p* < 0.01).

During the internalization of virions, the outer-capsid protein *σ*B may act as a protector protein for endosomal membrane-penetration protein *μ*B. Prior to *μ*B cleavage and rearrangement, *σ*B is degraded by proteases in endosomes before the exposure cleavage site of *μ*B. The proteolysis kinetics of outer-capsid protein *σ*B were assessed both in the presence and absence of 25HC. DF-1 cells were pretreated with 5 μM 25HC or mock treated for 12 h, followed by incubation with 100 MOI ARV at 4°C for 1 h. At 0, 60, 90, 120, or 150 min after adsorption, the *σ*B protein levels in cell lysates were assayed by Western blotting. As exhibited in Figure 6A, the *σ*B degradation occurred at 90 min and new synthesis began at 120 min in control cells. In comparison, the *σ*B became significantly degraded at 150 min in 25HC pre-treated cells. We also examined whether 25HC pre-treatment affects ARV attachment. Equal amounts of ARV were added to the mock- or 25HC pre-treated cells, and further incubated at 4°C for 1 h. The virions attached to the cells were determined by TCID_50_. As shown in Figure 6B, no differences were observed between 25HC-treated (5 μM) and mock-treated ARV infection groups. The 25HC pre-treatment had no significant effect on ARV attachment to target cells. Thus, it is plausible that 25HC disrupts the normal membrane physiology to delay the outer capsid proteolysis of virions, thereby delaying the generation of sites of origin for ARV replication.

### 3.6. CH25H Blocks cell–cell membrane fusion induced by the p10 protein of ARV

**Figure 7:**
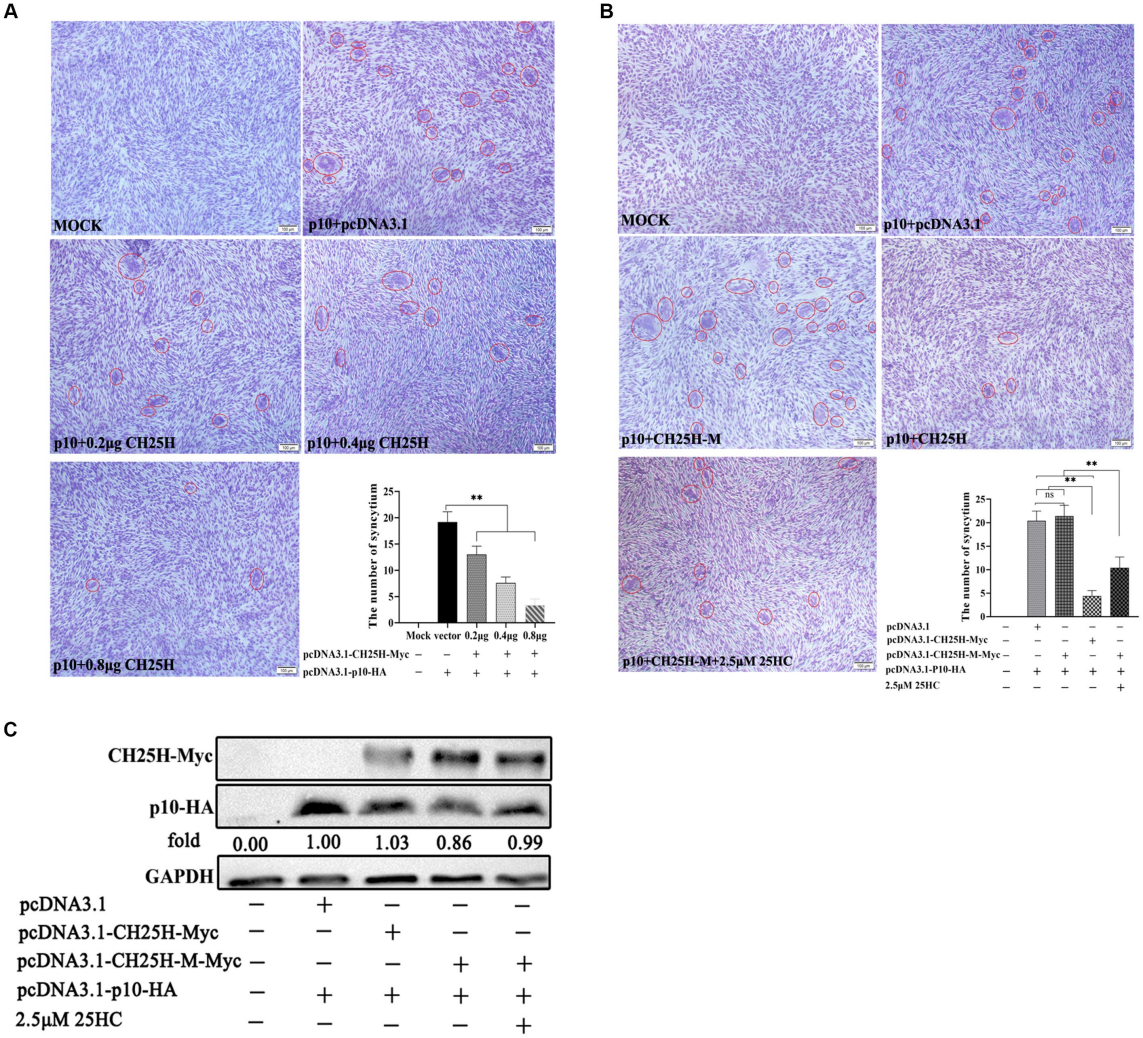
CH25H blocks cell–cell membrane fusion induced by the p10 protein of ARV. DF-1 cells were co-transfected with expression vectors encoding Myc-tagged chCH25H or chCH25H mutant and HA-tagged ARV p10 protein. The red circles indicate syncytia. The level of syncytium formation was detected by Giemsa-staining. Images were captured on an inverted microscope at 10× objective. The number of syncytia was determined by the average number of syncytia from five random fields using a × 10 objective. **(A)** CH25H blocks the cell–cell fusion induced by p10 of ARV in a dose-dependent manner. **(B)** The chCH25H mutant failed to reduce syncytium formation induced by ARV p10, and the extra addition of 25HC sufficiently complemented the lost activity of the chCH25H mutant. **(C)** The levels of wild-type chCH25H, enzymatic mutant chCH25H and ARV p10 protein were analyzed by Western blotting. Results are presented as means ± SD from three independent experiments. Significance was determined by Student’s *t-*test (ns, not significant; *, *p* < 0.05; **, *p* < 0.01).

Since cell–cell membrane fusion induced by ARV p10 protein is not linked to viral entry, we set up an *in vitro* cell-to-cell fusion assay based on p10 expression in DF-1 cells, independent of virus infection. Quantitative analysis of syncytia indicated that overexpression of wild-type chCH25H significantly inhibited the p10-induced syncytium formation in a dose-dependent manner (Figure 7A). Despite similar protein levels of p10, the overexpression of the mutant chCH25H failed to reduce syncytium formation induced by ARV p10. There was no significant difference in the number of syncytia between the mutant group and the empty vector group, while the relative number of syncytia in the mutant chCH25H group was significantly higher than those in the wild-type chCH25H group. Furthermore, the addition of 25HC sufficiently complemented the lost activity of the chCH25H mutant in blocking syncytium formation (Figures 7B,C). These results indicated that CH25H blocked cell–cell membrane fusion induced by the p10 protein of ARV, and that its inhibitory ability was dependent on its hydroxylase activity.

## 4. Discussion

ARV is an important pathogen that can result in substantial economic losses in the poultry industry. Despite current preventive and control strategies, efficient control remains difficult to achieve (Jones, 2000). Recent studies have demonstrated that ISGs, induced by IFN produced in the innate immune system, can inhibit a variety of viruses at different stages of the viral replication cycle. These studies provide new insights into the development of broad-spectrum antiviral therapies (Sen and Sarkar, 2007; Poynter and DeWitte-Orr, 2016; Raniga and Liang, 2018). One such ISG, CH25H, contributes to antiviral innate immunity by converting cholesterol to 25HC and regulating cholesterol and lipid metabolism (Zhao et al., 2020; Mao et al., 2022). CH25H and 25HC have been shown to exhibit broad antiviral activity against a variety of viruses (Liu et al., 2013; Zhao et al., 2020). For instance, CH25H has been identified as an antiviral host factor that restricts SARS-CoV-2 infection. Internalized 25HC produced by CH25H accumulates in the late endosomes and potentially restricts SARS-CoV-2 spike protein catalyzed membrane fusion by blocking cholesterol export (Zang et al., 2020). CH25H has also been implicated in inhibiting HIV replication by inducing the production of 25HC in infected cells, which intervenes in metabolic and infectious processes, regulates cholesterol homeostasis, and influences viral entry into host cells (Liu et al., 2013; Saulle et al., 2020). Shrivastava-Ranjan et al. revealed a previously unrecognized role of CH25H in inhibiting LASV glycoprotein glycosylation and reducing infectious virus production (Shrivastava-Ranjan et al., 2016). Furthermore, it has been reported that HCV infection triggers the up-regulation of interferon-inducible CH25H *in vivo*, and 25HC primarily restricts HCV at the RNA replication level by inhibiting the formation of the viral replication factory (Kusuma et al., 2015). Recently, Xie et al. demonstrated that CH25H inhibits ALV-J replication by promoting cellular autophagy (Xie et al., 2019, 2023).

Although a recent study has screened some ISGs against ARV infection and investigated the antiviral activity of IFIT5, the potential of CH25H to inhibit ARV replication remains unknown (Wang S. et al., 2022). To date, most literature describes the antiviral activities and the mechanism of CH25H against enveloped viruses, primarily by inhibiting virus-cell membrane fusion. Due to the lack of outer lipid membranes, the mechanisms underlying the protective effects of CH25H or 25HC against non-enveloped viruses are rarely reported. Of note, ARV is one of the only known non-enveloped viruses that cause cell–cell fusion. Intriguingly, our previous studies have indicated that cellular cholesterol in lipid rafts is crucial for ARV replication during both the entry stage and the post-entry stages (Duncan, 2019; Wang Y. et al., 2020). It could be speculated that CH25H may exhibit similar antiviral activities against ARV as it does against enveloped viruses. Nevertheless, whether ARV infection can induce the upregulation of chCH25H in target cells and whether chCH25H or 25HC restricts ARV infection remains unknown.

CH25H can be rapidly induced in a variety of mammalian tissues including liver, heart, muscle, brain, kidney and lung. Tissues with resident macrophage populations (liver, lung, and brain) present the highest levels of CH25H induction (Bauman et al., 2009). Another research found that type I interferons regulate CH25H expression in DCs and macrophages in mice (Park and Scott, 2010). However, the expression of CH25H varies in response to various viral infections. For example, after infection with viruses such as ZIKV, VSV and HIV, CH25H expression is significantly upregulated in host cells, whereas after infection with viruses such as PRRSV and HSV, CH25H expression is significantly downregulated. This may be due to different mechanisms of regulation of CH25H expression after infection with different viruses (Zhang et al., 2019; Zhao et al., 2020). In this study, we first analyzed the kinetics of endogenous chCH25H expression after ARV infection in a chicken macrophage cell line (HD11 cells). The expression of IFNA, IFNB, and chCH25H were all significantly upregulated at the early stage of infection. This pattern is consistent with previous reports about IFN production and CH25H following MRV infection in mammalian cell lines (Doms et al., 2018). Consequently, we propose that ARV infection triggered the induction of expression of type I interferons, which was followed by the rapid expression of chCH25H during the innate immune response. Beyond that, we found that 25HC treatment in DF1 cells significantly inhibits ARV replication in a dose-dependent manner at the entry and post-entry stages. Overexpression of chCH25H dramatically decreased ARV replication, and the knockdown of chCH25H by short interfering RNA (siRNA) promoted ARV infection. Herein, we demonstrate for the first time that both CH25H and its enzymatic production 25HC significantly inhibit ARV infection and replication. Therefore, 25HC has the potential as a natural antiviral agent to combat ARV.

Apart from antiviral mechanisms depending on enzyme activity, CH25H can also inhibit the infection of some viruses via a hydroxylase-independent mechanism (Ke et al., 2017; Lv et al., 2019; Li et al., 2020; Zhu et al., 2021). For instance, Ke et al. found that CH25H-M inhibits PRRSV replication not only through enzyme activity but also through the degradation of PRRSV nsp1α. Zhu et al. demonstrated that the CH25H mutant, which lacks hydroxylase activity, still restricts *Senecavirus* A infection (Ke et al., 2019; Zhu et al., 2021). In this study, a chCH25H mutant (H242Q and H243Q), which lacks hydroxylase activity and is unable to produce 25HC, was constructed to investigate whether the inhibitory effect of CH25H on ARV infection depends on its enzyme activity in DF-1 cells. Our results showed that the hydroxylase-inactive chCH25H mutant was unable to suppress ARV infection. Additionally, the *μ*NS and *σ*C mRNA levels, as well as *μ*NS protein expression of the chCH25H mutant group are even higher than those of the empty vector group. As a result, we proved that CH25H suppressed ARV infection by producing 25HC through an enzyme activity-dependent manner.

Cholesterol homeostasis is crucial for the infection of multiple viruses (Reboldi and Dang, 2018; Foo et al., 2022; Lee and Bensinger, 2022). Doms et al. proposed that 25HC altered cholesterol dynamics in a way that disrupted endosomal membrane physiology, limiting MRV particle trafficking and uncoating in the endosomes (Doms et al., 2018). Another example is the Niemann-Pick C1 (NPC1), which is a transmembrane protein that mediates the exportation of cholesterol from late endosomes and lysosomes. According to a recent study, NPC1-mediated cholesterol transport is required for MRV penetration from the late endosome into the cytoplasm, but not for MRV attachment, internalization, or uncoating (Ortega-Gonzalez et al., 2022). In the present study, we preliminarily investigated the potential inhibition mechanism of 25HC against ARV at the viral entry stage. 25HC pre-treatment significantly inhibited ARV replication at the entry stage in a dose-dependent manner. 25HC also postpones ARV outer-capsid protein *σ*B proteolysis, but it does not prevent ARV attachment. The results are consistent with previous observations of MRV uncoating being restricted by 25HC. It is postulated that 25HC alters the cholesterol content of membranes in endocytic compartments, leading to a delay in the proteolytic processing of *σ*B protein. Accordingly, exposure of the hydrophobic conformer of the *μ*B protein is delayed, and the efficiency of ARV infection is decreased. However, the detailed mechanisms should be evaluated in a future study.

Another interesting issue is the unusual fusogenic ability of ARV. It is widely accepted that the enveloped viruses cause virus-cell membrane fusion during viral entry, fusing their virion envelope with the host membrane during viral entry (Helenius, 2018). Consequently, non-enveloped viruses do not enter cells through membrane fusion, due to their lack outer lipid membranes. However, an exception to this is fusogenic reoviruses. Among non-enveloped viruses, only a few reoviruses (ARV, Nelson Bay reovirus, baboon reovirus, reptilian reoviruses, and aquareoviruses) can cause cell–cell membrane fusion to form syncytia (Shmulevitz and Duncan, 2000; Salsman et al., 2005). Fusogenic reoviruses encode viral fusogens termed fusion-associated small transmembrane (FAST) proteins (Ciechonska and Duncan, 2014). These reovirus FAST proteins are expressed intracellularly as non-structural proteins and transported to the plasma membrane. Unlike enveloped viruses, fusogenic reoviruses cause host cellular membrane to fuse from within the infected cell. Although not necessary for viral entry, the fusion mediated by FAST proteins is involved in cell–cell transmission and release of fusogenic reoviruses (Duncan, 2019). Besides that, a recent study using reverse genetics systems found that cell–cell fusion mediated by FAST proteins enhances the replication and pathogenicity of non-enveloped dsRNA viruses (Kanai et al., 2019). Consequently, it is of interest to investigate whether CH25H blocks cell–cell membrane fusion induced by p10. To this end, we evaluated the effect of chCH25H expression on ARV p10 FAST protein-mediated membrane fusion based on the expression of p10 in DF-1 cells, independent of virus infection. Our data showed that wild-type chCH25H expression significantly reduced syncytium formation mediated by p10 protein, whereas the overexpression of mutant chCH25H failed to reduce syncytium formation induced by p10. Moreover, both wild-type and mutant chCH25H overexpression did not affect p10 expression. Of note, our previous study indicated that p10 protein accumulated in cholesterol-rich lipid rafts of cellular membrane and assembled multimerically to trigger the fusion process (Wang Y. et al., 2020). Hence, we speculated that 25HC catalyzed by CH25H can disrupt the normal membrane physiology of cholesterol-rich lipid rafts to prevent p10-induced fusion, probably via altering cholesterol content, spacing of phospholipids, or membrane curvature of lipid bilayers.

In summary, we have demonstrated for the first time that ARV infection triggers the induction of the interferon-stimulated protein CH25H at the early stage of infection, and CH25H suppresses ARV replication by its product 25HC, depending on its enzyme activity. We Also found that 25HC likely inhibits viral entry by delaying the kinetics of ARV uncoating. It also restricts virus release and dissemination by depleting accessible cholesterol on the plasma membrane to block cell–cell fusion. These results provide new insights into the role of lipids in the innate immune response to ARV infection, as well as the pathogenesis and control of ARV.

## Data availability statement

The original contributions presented in the study are included in the article/Supplementary material, further inquiries can be directed to the corresponding author.

## Author contributions

YaW and YuW conceived and designed the experiments. WZ performed the research and analyzed the data. YuW and WZ wrote the paper. YZ carried out additional analyses. ZB, CZ, XZ, and YaW contributed to refining the ideas and finalizing this paper. All authors contributed to the article and approved the submitted version.

## Funding

This work was supported by the National Natural Science Foundation of China (31902250), the China Agriculture Research System of MOF and MARA (CARS-40), and a project funded by the Priority Academic Program Development of Jiangsu Higher Education Institutions (PAPD).

## Conflict of interest

The authors declare that the research was conducted in the absence of any commercial or financial relationships that could be construed as a potential conflict of interest.

## Publisher’s note

All claims expressed in this article are solely those of the authors and do not necessarily represent those of their affiliated organizations, or those of the publisher, the editors and the reviewers. Any product that may be evaluated in this article, or claim that may be made by its manufacturer, is not guaranteed or endorsed by the publisher.
