# Supplementary material for: Cholesterol 25-hydroxylase suppresses avian reovirus replication by its enzymatic product 25-hydroxycholesterol
Source: Front Microbiol. 2023 Jun 29;14:1178005. doi: 10.3389/fmicb.2023.1178005 (PMC10340090; doi:10.3389/fmicb.2023.1178005)
Supplement: Supplementary file 1 [file Data_Sheet_1.docx]

Supplementary Material

1. **Supplementary Figures**

**Supplementary Figure 1.** The poly (I:C) treatment upregulates the mRNA levels of IFNA, IFNB and chCH25H in HD11 cells and DF-1 cells. (A) The HD11 cells were treated with poly (I:C) (2 μg/mL), and the mRNA levels of chCH25H, IFNA and IFNB were determined by qRT‐PCR at the indicated time points (B) The DF-1 cells were treated with poly (I:C) (2 μg/mL), and the mRNA levels of chCH25H, IFNA and IFNB were determined by qRT‐PCR at the indicated time points. Results are presented as means ± SD from three independent experiments. Significance was determined by Student’s *t-*test (*, *P* < 0.05; **, *P* < 0.01).

**Supplementary Figure 2.** The knockdown efficiency of siRNA1, siRNA2, and siRNA3 targeting chCH25H. The mRNA levels of CH25H were determined by qRT‐PCR. Results are presented as means ± SD from three independent experiments. Significance was determined by Student’s *t-*test (*, *P* < 0.05; **, *P* < 0.01).

**Supplementary Figure 3.** The chCH25H mutant (H242Q and H243Q) is unable to produce 25HC. 25HC was quantified by ELISA. Results are presented as means ± SD from three independent experiments. Significance was determined by Student’s *t-*test (ns, not significant, *, *P* < 0.05; **, *P* < 0.01).
