# Supplementary figures and images for: Cholesterol 25-hydroxylase suppresses avian reovirus replication by its enzymatic product 25-hydroxycholesterol
Source: Front Microbiol. 2023 Jun 29;14:1178005. doi: 10.3389/fmicb.2023.1178005 (PMC10340090; doi:10.3389/fmicb.2023.1178005)

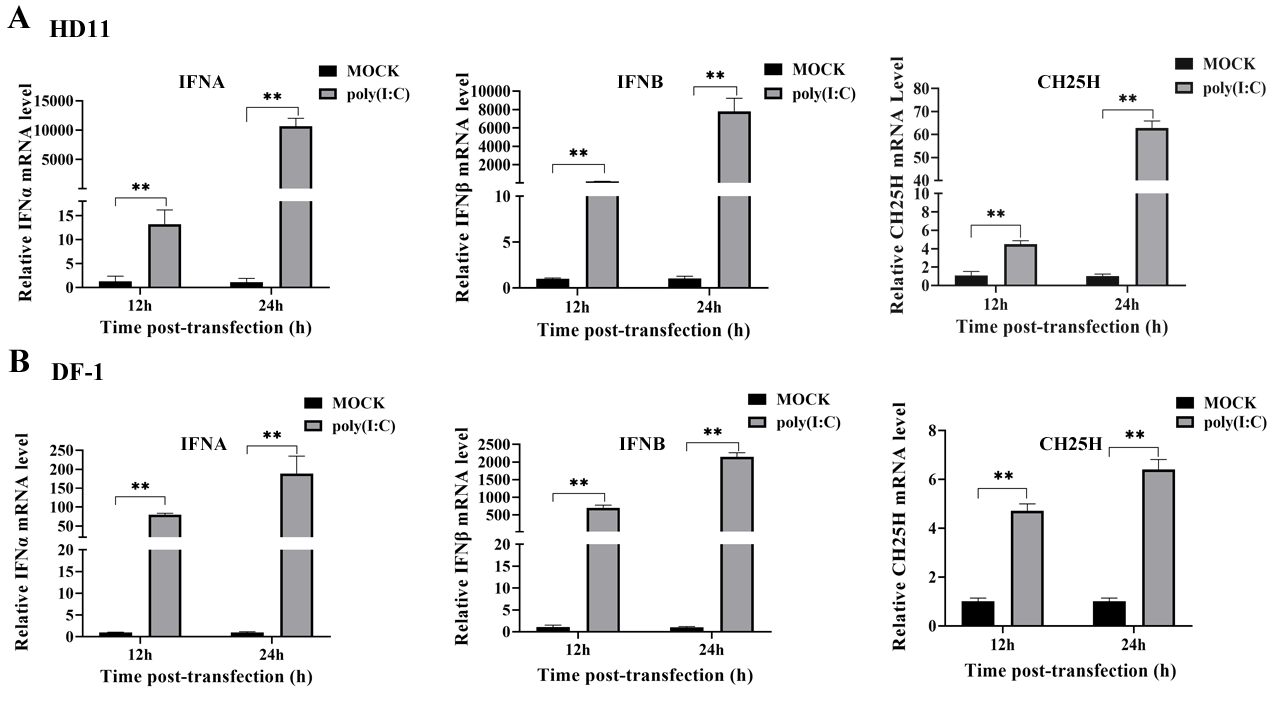

Supplement: Supplementary file 2 [file Image_1.TIF]
